# Indents

**Published:** 1908-09-15

**Authors:** 


### INDENTS.

☞ **Brief Articles of Technical or Scientific Value will receive notice and comment. Contributions invited**

**Dentists Honor Dr. H. M. Reid.** Members of the faculty of the college of dentistry at the University of Minnesota gave a dinner, June 5, in honor of Dr. H. M. Reid. Dr. Reid has decided to give up his active practice, and he will take up his residence in New Jersey. Dr. Reid has been connected with the dental department of the university for several years. He has been secretary and treasurer of the Minnesota Dental Society for the last twenty-five years. Dr. Reid was a well-known practitioner in Cincinnati for many years before he went to Minneapolis.

**To Remove Rust from Instruments.** Pharmaceutische Centralblatt prints an effective process for removing rust from surgical instruments. The instruments are placed over night in a saturated solution of stannous chloride, which causes the spots to disappear by reduction. The articles are then rinsed in water, laid in a hot solution of soda soap, and dried. It is well to rub them with absolute alcohol and prepared chalk. Another convenient method for removing rust is to lay the instruments in kerosene. Paraffine oil is the best preservative against rust, and the most convenient way of applying it without getting an unnecessary thick coating is as follows: One part of the oil is dissolved in two hundred parts of benzine, and the objects, after being thoroughly dried, and warmed, are plunged into the solution. Instruments with joints, as scissors or needle holders are washed in the fluid, in order to cause it to penetrate into all crevices, and the benzine is then allowed to evaporate in a dry room.—*British Dental Journal.*

**Treatment of Pyorrhea.** I do not deny that there is a constitutional element present, but that that constitutional element constitutes pyorrhea and that pyorrhea cannot be cured without its elimination does not appeal to my reason.

The roots must be scaled thoroughly, success is out of the question otherwise, but in my opinion this should be done with as little laceration or destruction as possible. No good can be accomplished by scraping the roots except for the removal of deposits and that in reality is not scraping but carefully cleaning off the deposits. Incidentally, the half-dead and effete matter will be removed by the instruments from the socket and flushed out by the attendant hemorrhage. If care is exercised and the work of removal performed thoroughly, I fail to see why any cauterization should be necessary, any more than after a general surgical operation.

Many have assumed such treatment to be necessary, but how many have tried the experiment of doing without it? During the entire period in which I have treated pyorrhea I have never used any form of treatment other than the scaling and flushing with antiseptics. The results have proved eminently satisfactory.

Experience has proven to me that if the deposits and all other foreign and dead matter have been thoroughly removed by instrumentation and flushing the sockets with an antiseptic, the tissues will heal and resume their normal tone if kept clean subsequently.

Of course there are exceptions where, in spite of any treatment short of extraction, a cure is impossible, but it is my opinion that better results con be obtained without the use of acids than with them and in less time.

As regards the rheumatic or gouty diathesis, there can be no doubt of its having a distinct bearing on pyorrhea in some cases, but it seems to me reasonable to believe that the part played by such systematic conditions has more to do with furnishing the material of which the deposits are formed than with causing their localization on the roots of teeth. Unless some local irritation brings about the precipitation of the urates we have no deposits.

This I believe to be true in regard to pericemental abscess. I do not know just how many such cases have come before me for treatment, but there have been quite a large number all told and in every case deposits have been present, and entirely independent of all connection with the gingival margin.

During periods of acute activity in the rheumatic diathesis there is a greater tendency toward inflammation, but I believe that if the teeth are thoroughly cleaned at frequent intervals, by the dentist, there will be no recurrence of the pyorrhea.—R. G. Hutchinson, Jr., in the *Journal* for June.

**A New Crown.** At least I call it new. I have never seen it mentioned in the dental journals. In making this crown in its simplest form, prepare the root as you would for a Davis or Logan crown with root well under gum labially. Then take a Steele facing without backing, no Steele backing is used, grind gingivally to shape. Now take take a square platinum pin, 16 or 18 gauge, and fit in root, bringing the pin a little toward the lingual side of root and allowing the pin to protrude about three-sixteenths of an inch from the root. The protruding end should be slightly flattened or notched. Now take inlay wax, after warming and kneading carefully, and press firmly over pin and down on root. Then take your prepared facing and press it to position, and with a warm spatula shape the wax in the mouth, approximately as you wish your backing to be. Cool with ice or cold water and remove; the pin should come away with your wax and facing. Then finish your waxing; if you wish, you may allow wax to extend slightly over incisal edge for protection. Replace on root to see that all is correct. Remove, invest and cast as you would an inlay. Heat your flask pretty well when casting. Twenty-karat solder may be used, or even platino-silver casting metal if you desire, which does no oxidize and casts at the same temperature as 22k gold.

This will make a pretty crown and a strong one, and can be made almost as quickly as one can tell about it.

If you desire a banded root crown, proceed with cap and pin as you would for a Richmond crown, only allow pin to protrude as in plain crown.

I have never had a facing break on one of these crowns, but if one does it can be easily and quickly replaced.

Dr. W. C. Dalbey, in *Summary*.

**Securing Patients.** "What is the secret of your life?" asked Elizabeth Barrett Browning of Charles Kingsley. "Tell me that I may have mine beautiful too."

"I had a friend," was the reply.

President Roosevelt could never have accomplished anything equal to what he has but for the powerful, persistent, enthusiastic assistance of his friends.

Every successful dentist owes his success largely to his friends. Just think what it means for a dentist to have enthusiastic friends always looking out for his interests, saying a good word for him at every opportunity, supporting him, speaking for him in his absence, when so many times a dentist needs a friend, stopping slanders, killing lies which might injure him, correcting false impressions about his work, overcoming the prejudices created by some accident or mishap or possibly some mistake or slip or a bad impression made at some silly moment; friends who are always doing something to give us a lift or help us along.

Dr. Marden, Editor of *Success*, gave to every dentist the key to advertising and building up a practice when he said: "If you are starting out in a profession and wanting patients, what more profitable way of occupying your spare time than in cultivating friendship?" The reputation of having a lot of staunch friends will give you backing and bring to you patients . It has been said, "Destiny is determined by friendships."

Make friends, but he who secures friends must show himself friendly. A host of true, loyal friends will multiply your natural ability many times. But listen, too often we fail to appreciate this true loyality of our friends and instead of giving them credit and thanking them for their kindness to us, we give ourselves credit for our great ability, smartness and shrewdness. Many older men have, I believe for this very reason, begun to lose their hold on a community simply because they have ceased to make new friends and failed to appreciate their old friends.—Dr. O. U. King, in August *Summary.*

**A Preliminary Report on the Use of a New Antiseptic Powder in the Treatment of Pyorrheal Pockets.**

In the treatment of pyorrheal pockets it is difficult to retain a liquid antiseptic in the spaces for any length of time, and thus a frequent application of the antiseptic is necessary. It has therefore been my endeavor to obtain an efficient antiseptic powder that would remain in *situ* and produce a continuous beneficial effect upon the diseased area. My experience demonstrates that methylene-disalicylic-acid-iodid (formidin) fulfils these requirements most perfectly. Its chemical formula is $C_{10}H_{10}O_6I_2$, and it is a compound derived from iodin, formic aldehyd, and salicylic acid. In the presence of alkaline secretions it decomposes, producing the characteristic reactions of its constituent parts. As formidin is tasteless and has a slight, rather pleasant odor, and as it is devoid of toxic effects, it can be employed with perfect safety and without any disagreeable symptoms ensuing.

I must admit that the number of cases in which I have employed this powder is not sufficient to absolutely demonstrate its value, but my results so far have been so specific that I here report one of my cases in detail, hoping that I may hear of others who have employed similar methods in treating this important disease.

*Case:* L. B. M. Female, age forty. Before this case came to me for treatment an upper second molar had been lost, and at the time of examination three remaining second molars and one third molar were very loose. These presented prominent open pockets with much irritation and soreness. A large amount of tartar was present and considerable absorption of the alveolar process had taken place. My treatment consisted of scaling the teeth to remove the tartar and thoroughly cleansing the pocktes with hydrogen dioxid. Thereupon, after drying the site and taking care to keep away moisture by means of napkins, I proceeded to pack the pockets with formidin. The packing was accomplished by means of a modified amalgam syringe carrier. The home treatment consisted of massage, of painting the gums with tincture of iodin, and of rinsing the mouth with alkaline and astringent washes. For constitutional effects the patient received alteratives and tonics and her diet was careful restricted. During the more acute stages the pockets were cleansed and packed, as described above, this treatment lasting four weeks with three visits each week. After this period the formidin was sealed in by means of a suitable retainer, and was changed, at first every week, and finally every two weeks, until cured.

The results of this treatment were eminently satisfactory both to the patient and to myself, and as formidin can be tolerated by the most sensitive patient without too frequent applications and without any disagreeable effects, I believe it to be one of the most efficient methods at our disposal for overcoming this condition.—H. Otis Logue, D.D.S., *Cosmos.*

**How to Stop Bleeding After Extractions.** According to the standards of modern surgery, rinsing of the mouth with water after extractions must be regarded as unhygienic. Numerous infections can be attributed to this obselete method. Although the observation of past generations that cold is a good hemostatic is quite correct, yet water of a temperature low enough to bring about the contraction of the blood vessels and a rapid formation of the coagulum should never be used in the mouth. Moreover, the contraction of the blood vessels and the formation of the thrombus is arrested rather than accelerated by irrigation. In most cases, the bleeding after extractions is, very small. If, however an abnormally situated artery in the maxillary ridge is lacerated or if there are aneurysms present in the proximity of the teeth extracted or if the patient be subject to hemophilia, the local application of water is absolutely inadequate.

After the extraction of a tooth with inflamed pulp, where the gums are not infected, the edges of the wound are pressed together and a pad of sterilized cotton is laid over the wound and held by the patient's bite until the bleeding has ceased. If the wound is infected by a previous periostitis, of if abundant bleeding is to be feared, a strip of iodoform gauze is packed into the wound and a pad of sterilized cotton inserted, which is again held by the patient until the bleeding has stopped. The tampon is removed after two or three days and in exceptionally large wounds renewed several times. This tampon will not only stop the bleeding, but will also prevent the premature closing of the edges of the wound and accelerate the formation of sound granulations.—Prof. Dr. Brandt, *Berliner Zahnaerztliche Halbmonatsschrift.*

[The foregoing argument of Prof. Dr. Brandt should not be taken to apply to the use of water in general, but only to its use within the limitations to which he refers. It should be generally known that hot water, *i. e.* from 115 to 130 degrees Fahrenheit is a more efficient hemostatic than is cold water, whereas water of about blood temperature or slightly above it produces a relaxing effect upon the arterial coats, causing dilatation and therefore increase of hemorrhage. Water of less than 115 degrees Fahrenheit thould therefore not be employed as a hemostatic. Injected from a syringe into a bleeding socket at a temperature as hot as can be borne by the patient, hot water is a most efficient hemostatic in case of post-extraction hemorrhage. Avoidance of infection from the water source is secured by first bringing the water to the boiling point and then reducing the temperature to the point above referred to before injecting it into the bleeding alveoli. The successful use of hot-water irrigations in the control of post-partum hemorrage and for arresting capillary hemorrhage in tissues after operation in localities where the ordinary surgical means for control of bleeding are not practicable, furnishes ample precedent for the use of hot water to arrest hemorrhage after extraction. Furthermore, its successful use by any dental practitioner has placed this method upon a sound basis of practical utility. It is noticeable that in extraction cases where there has been a previous periostitis, healing takes place rapidly after the hot-water treatment, and there is a remarkably quick subsidence of all inflammatory symptoms.—*Cosmos*.]

**Cement Solution in Acid Saliva.** After all that has been said and done, the fine cement film of an inlay is no protection against disintegration; it only looks better, especially if the margins of the inlay tend to discolor. This explains why the old Bing gold inlay lasts so well, consisting as it does of a thin gold shell and pin cemented on to a cavity. Badly fitting though the edges have been, I have seen Bing inlays last for three years and do not remember ever having been able to pull one out.

My tests lead to the following conclusions: (1) In saliva solutions of acid, cement ordinarily dissolves more readily than enamel, and (2) some salivas are able to protect cement from decalcification and some salivas are not.

Where decalcification occurs as the only factor, a fine line of cement will dissolve more rapidly than a coarse line of cement; but where friction and the jamming of the carbohydrates into a coarse line by mastication occurs, undoubtedly the cement in a coarse line will disappear more rapidly than in a fine line. But since a fine line of cement looks better than a coarse line, the extreme edges of the inlay should be as perfectly adapted as possible; but just back of the edges there should be a large thick body of cement that will control for years any slight solvent that may chance to get past the close-fitting edges. In plain words, the inlays should fit perfectly and then care should be taken to undercut both cavity and inlay to that the cement may act as a dowel and as a neutralizer of penetrating acid.

When the gold inlay is on the grinding surface, mastication will no doubt often swage the edges of the metal into apposition to the enamel as dissolution of the cement line takes place. But what I have often said before I now repeat: The best inlay consists of a gold filling set in cement where the edges have been finally cleaned of cement and fresh gold hammered or burnished into accurate apposition to the margins.

I finally chose lactic acid for my testing solution, not because acetic, citric, butyric, and valeric acids might not also attack the cement in the mouth, but because its action in my tests more nearly represented the kind of decalcification which I had noted in the mouth. Valeric and butyric acids slightly attack Harvard cement, but acetic and citric acids do it quite vigorously, and I have often wondered if these acids were not responsible for the dark cement line that sometime appears around inlays in the front teeth, these acids being not products of fermentation but ingredients of food.

I did not speak of the effect of acid calcium phosphate or acid sodium phosphate because these, as would be expected do not attack cement, however much they may attack tooth-enamel.

Before closing, let me say that I am putting in more inlays now than I ever did. I believe in the future of the inlay more than ever, and I hope some time to be able to find some means by which the saliva may be modified so that its power of resisting decalcification may be increased. Whether the sulfocyanate of potassium or the phosphates of soda and calcium are to be credited with this acid-restraining power, or whether this power is purely a property of saliva as a living fluid, will in time probably be demonstrated. —Jos. Head, in August *Cosmos*.

**Oil Polish in Prophylaxis.** The author suggests the use of oil prophylaxis in combating caries in its incipiency or its very beginnings, to sterilize the teeth and to render them as immune as possible to the attacks of caries. Especially the approximal surfaces of the teeth, the sulci, fissures and the labio-buccal necks must be protected. To accomplish this purpose the author recommends a polishing, in connection with an oil impregnation, at the hands of the dentist every three or six months, according to age and condition of the teeth, and a daily brushing with oil-paste by the patient.

For this oil-polish Dr. Kleinsorgen employs the following method: The teeth are carefully examined, every cavity is treated and filled, every trace of tartar removed and every visible roughness ground off smoothly. Fine precipitated carbonate of lime is mixed with vaseline oil into a stiff oil-paste, which is applied by means of ordinary rotary brushes. The polish itself is applied in five sections, beginning with the upper left series of teeth, then the lower left, the upper right, the lower right, and finally the six front teeth. For the sake of thoroughness cofferdam is applied at two different sittings. The teeth are first thoroughly dried with cotton and hot air. Special attention is to be given to fissures and approximal surfaces, especially of the front teeth. In order to diagnose very fine fissures, which are due to imperfect calcification of the enamel, especially delicate exploring probes are used; these fissures are enlarged with very fine burs and filled. the upper incisors are sufficiently separated by means of flexible separating files, and the approximal surfaces are polished first with various grades of sandpaper disks, and finally with paper disks well saturated with oil. Filling of even the most minute fissures in the enamel, including the foramina caeca or lingual pits of the incisors, even though no caries or discoloration is visible, and sufficient separation of the incisors, including the space between canine and first premolar, are primary conditions for a successful prophylaxis. In this way the chances of preservation of the teeth are improved by at least fifty percent.

A patient willing to undergo the oil treatment several times a year and to make one daily application of oil-paste has the guarantee of never being affected by caries of the necks of his teeth, in the fissures of his molars, or in the spaces between incisors and the first premolar. The author advises a quarterly application for children, vegetarians, bakers and confectioners, also during pregnancy and lactation and semi-annual application for all other patients. As a means for securing what he terms a natural separation of the molars, the author recommends the extraction of all second molars soon after eruption, or of the first molars shortly before eruption of the second molars. The oil prophylaxis promises to do away entirely with the patient's fear of the dentist's operating room.

While the method as above suggested by Dr. Kleinsorgen has certain features which seem to entitle it to thoughtful consideration, his suggestion with reference to filing the interproximal surfaces of contact or of securing natural separation by the extraction of molars will certainly be condemned by every right-thinking practitioner, and especially by those who have had opportunity to witness the devastating ravages of such a method, which characterized the practice of many operators a little over a generation ago. At that time men believed in the theory that lateral pressure of the teeth was one of the causes of caries, and acting on that theory, produced permanent separations between the teeth in various ways, all of which resulted in an enormous destruction of teeth from disease of their sockets, directly induced by the pernicious separation method.—*Cosmos.*—By Dr Kleinsorgen, in *Archiv fuer Zahnheilkunde.*

